# The impact of COVID-19 pandemic on trends in the recorded incidence of Long-Term Conditions identified from routine electronic health records between 2000 and 2021 in Wales: a population data linkage study.

**DOI:** 10.23889/ijpds.v7i3.2011

**Published:** 2022-08-25

**Authors:** Cathy Qi, Tim Osborne, Rowena Bailey, Joe Hollinghurst, Ashley Akbari, Alison Cooper, Ruth Crowder, Holly Peters, Rebecca-Jane Law, Anthony Davies, Ruth Lewis, Mark Walker, Adrian Edwards, Ronan Lyons

**Affiliations:** 1 Swansea University; 2 Cardiff University; 3 Welsh Government; 4 Bangor University

## Objectives

The COVID-19 pandemic has resulted in delayed diagnosis and treatment for cancer patients and increases in elective surgery waiting lists. The impact on other ‘long-term’ conditions (LTCs) is unclear. We examined the effects of the pandemic on the recorded incidence of 20 LTCs to inform decisions on treatment pathways and resource allocation.

## Approach

We included Welsh residents diagnosed with any of 20 LTCs for the first time between 2000-2021.

Data were accessed and analysed within the Secure Anonymised Information Linkage (SAIL) Databank.

The primary aim was to assess the impact of the COVID-19 pandemic on trends in recorded incidence. Secondarily we examined incidence by socio-demographic and clinical subgroups: age, sex, deprivation quintile, ethnicity, frailty score and learning disability.

Incidence were presented as monthly rates for each LTC. We performed interrupted time series analyses to estimate; the immediate and long-term change in rates following the pandemic; and the size of the undiagnosed population.

## Results

We included 2,206,070 individuals diagnosed with at least one LTC.

An immediate reduction in recording of new diagnoses was observed in April 2020 across all 20 LTCs, followed by a gradual recovery towards pre-pandemic levels over the next 18 months, though at different rates across conditions. The largest difference between observed and expected (as predicted using pre-pandemic trends) incidence between January 2020 and June 2021 were in the diagnoses of COPD (-43%, 95% CI (-50%, -34%)), Asthma, Hypertension and Depression and the smallest difference was in Type 1 diabetes, dementia, stroke and TIA (-8%, 95% CI (-19% ,5%)).

Differences in the proportions of incidence by socio-demographic and clinical subgroups in the years preceding and following the pandemic have also been analysed (results to be finalised).

## Conclusion

There was an abrupt reduction in the observed incidence of all 20 LTCs after March 2020 followed by a gradual recovery over consequent months towards pre-pandemic levels. Of 20 LTCs, 15 strongly indicate a reservoir of yet undiagnosed patients. The results from this study will have implications in resource allocation.

